# Resveratrol Prevention of Diabetic Nephropathy Is Associated with the Suppression of Renal Inflammation and Mesangial Cell Proliferation: Possible Roles of Akt/NF-**κ**B Pathway

**DOI:** 10.1155/2014/289327

**Published:** 2014-02-09

**Authors:** Feng Xu, Yuehui Wang, Wenpeng Cui, Hang Yuan, Jing Sun, Man Wu, Qiaoyan Guo, Lili Kong, Hao Wu, Lining Miao

**Affiliations:** ^1^Department of Nephrology, The Second Hospital of Jilin University, 218 Ziqiang Street, Changchun 130041, China; ^2^Department of Cardiology, The Second Hospital of Jilin University, 218 Ziqiang Street, Changchun 130041, China

## Abstract

The present study was to investigate the protection of resveratrol (RSV) in diabetes associated with kidney inflammation and cell proliferation. Rat mesangial cell and streptozotocin-induced type 1 diabetes mouse model were used. *In vitro*, RSV attenuated high glucose-induced plasminogen activator inhibitor (PAI-1) expression and mesangial cell proliferation, as well as Akt and nuclear factor-kappa B (NF-**κ**B) activation. The similar results were recaptured in the experiment with Akt inhibitors. *In vivo*, mice were divided into three groups: control group, diabetes mellitus (DM) group, and RSV-treated DM group. Compared with control group, the kidney weight to body weight ratio and albumin to creatinine ratio were increased in DM group, but not in RSV-treated DM group. Furthermore, the increased expression of PAI-1 and intercellular adhesion molecule-1 in diabetic renal cortex were also reduced by RSV administration. Besides, the kidney p-Akt/Akt ratio and NF-**κ**B were significantly increased in DM group; however, these changes were reversed in RSV-treated DM group. Additionally, immunohistochemistry results indicated that RSV treatment reduced the density of proliferating cell nuclear antigen-positive cells significantly in glomeruli of diabetic mice. These results suggest that RSV prevents diabetes-induced renal inflammation and mesangial cell proliferation possibly through Akt/NF-**κ**B pathway inhibition.

## 1. Introduction

Nowadays, diabetic nephropathy (DN) has become a serious problem worldwide because of its rapidly increasing rates, as well as economic and social burden. Unfortunately, the intimate mechanisms leading to the development and progression of this disease are complex and not yet fully understood [1].

Glomerular mesangium expansion is one of the characters of early DN. Accumulated data suggest that the predicted evolution of diabetic glomerulopathy is comprised of an early, transient mesangial cell proliferation and subsequent hypertrophy of these cells that herald the slow progression into glomerulosclerosis [2]. In addition, inflammation is also an important pathophysiological factor in the development and progression of DN [3, 4]. Recent studies have emphasized the critical roles of inflammatory response in development of DN [5, 6]. Different inflammatory molecules, including chemokines, adhesion molecules, and proinflammatory cytokines, may be critical factors involved in DN.

Resveratrol (RSV) is a phytoalexin polyphenolic compound found in various plants, such as grapes, nuts, and berries. What is more, the number of plants involving this compound is growing [7]. A series of potential beneficial effects of RSV should be attributed to its multiple bioactivities. Function of RSV has been extensively explored for its powerful antioxidant capacity and specific effects on proteins and/or signaling cascades, such as Sirt1, adenosine monophosphate activated kinase, phosphatidylinositol-3 kinase (PI3K)/Akt, and JNK/nuclear factor-kappa B (NF-*κ*B) in DN both *in vivo* and *in vitro* [8–10]. By using 12-week old *db*/*db* mice, Kim et al. found that RSV decreased the activity of PI3K/Akt phosphorylation, resulting in a decrease in BCL-2-associated X protein (BAX) and increases in BCL-2 and superoxide dismutase production in diabetic kidney [8]. Additionally, Zhang et al. demonstrated that RSV prevented high glucose-induced kidney mesangial cell proliferation and fibronectin expression through inhibition of high glucose-induced JNK and NF-*κ*B activation, NADPH oxidase activity elevation, and reactive oxygen species production [10]. However, whether there is a direct link between Akt and NF-*κ*B for the protection of RSV from DN was not addressed in these two separate papers. In the present study, therefore, we aimed to determine whether RSV treatment attenuated renal inflammation and mesangial cell proliferation under diabetic condition both *in vivo* and *in vitro*. By using Akt activity inhibitors, we have mechanistically defined whether the protective effect of RSV on DN was due to Akt-dependent depression of NF-*κ*B.

## 2. Research Design and Methods

### 2.1. Rat Mesangial Cell (RMC) Culture and Treatment 

RMCs were cultured in Dulbecco's modified Eagle medium (DMEM; Thermo Scientific Hyclone, Beijing, China) containing 5.6 mM glucose (normal glucose, NG), 10% Fetal Bovine Serum (FBS, Thermo Scientific Hyclone, Beijing, China), 100 U/mL penicillin (Thermo Scientific Hyclone, Beijing, China), and 100 *μ*g/mL streptomycin (Thermo Scientific Hyclone, Beijing, China). RMCs were exposed to 25 mM D-glucose (high glucose, HG) with 0.2% bovine serum albumin (BSA) and 0.5% FBS for 10 min–48 h. D-mannitol (19.5 mM) was used as a hyperosmotic control. LY294002 (LY, 10 *μ*M, Sigma-Aldrich Co., St. Louis, MO, USA), MK-2206 (MK, 1 *μ*M, Selleck Chemicals Co., Houston, TX, USA), or RSV (25 *μ*M, Sigma-Aldrich Co., St. Louis, MO, USA) dissolved in dimethyl sulfoxide (DMSO) was added. Cells were harvested at the indicated times. Akt (Cell Signaling Technology, Danvers, MA, USA), phospho-Akt (p-Akt, Ser473, Cell Signaling Technology, Danvers, MA, USA), NF-*κ*B p65 (Cell Signaling Technology, Danvers, MA, USA), plasminogen activator inhibitor (PAI-1, Abcam Inc., Cambridge, MA, USA), and *β*-Actin (Cell Signaling Technology, Danvers, MA, USA) expression were determined by Western blotting assay.

### 2.2. Cell Proliferation Assay

Cells were seeded into 96-well plates at a proper density. When the confluence reached at 60%–70%, the medium was replaced with DMEM containing NG (5.6 mM) and 0.2% BSA. 24 h later, the cells were pretreated with 10 *μ*M LY294002, 1 *μ*M MK-2206, or an equal volume of DMSO for 30 min and then incubated for another 24 h with or without HG (25 mM) in the presence or absence of RSV (25 *μ*M). Cell proliferation was determined by Cell Counting Kit-8 (CCK8, Beyotime, Shanghai, China) or by bromodeoxyuridine (Brdu) incorporation using the cell proliferation ELISA kit (Roche, Mannheim, Germany) according to manufacturer's procedures.

### 2.3. Experimental Animals

Male FVB mice at eight weeks of age and 26–30 g weight, were purchased from Vital River Laboratory Animal Technology Co. Ltd. and housed in Jilin University Animal Center under standard vivarium conditions (22°C, 12 h light/dark cycle) with free access to water and standard rodent chow. The animals were acclimatized to the laboratory conditions for 2 weeks prior to the inception of experiments. All animal procedures were approved by the University Animal Care and Use Committee, which is certified by the Chinese Association of Accreditation of Laboratory Animal Care.

### 2.4. Induction of Experimental Models

Mice were randomly divided into three groups (each group contains at least 6 mice): control group, diabetes mellitus (DM) group, and RSV-treated DM group. Experimental diabetes was induced with multiple low doses of streptozotocin (STZ). Mice were injected intraperitoneally with STZ (Sigma-Aldich, St. Louis, MO, USA), which was freshly dissolved in cold citrate buffer (pH 4.5), at a concentration of 50 mg/kg daily for 5 consecutive days. And control mice received multiple injections of the same volume of sodium citrate buffer. Five days after the last injection, mice with moderate diabetes (i.e., blood glucose concentration ≥14 mM, 3 consecutive days) were selected for the experiment. RSV-treated DM group was given RSV by oral gavage in a dose of 10 mg/kg/day for 12 weeks. At the same time, both control and DM groups were given an equivalent amount of saline by oral gavage for the same period. The dosage was adjusted every week based on any change in body weight during the whole period of study. After 12-week treatment with RSV or saline, the mice were fasted overnight, anaesthetized, and killed by cervical decapitation.

### 2.5. Mouse Urinary Albumin to Creatinine Ratio (ACR) Detection

Spot urine was collected before mice were killed. Urinary albumin and creatinine excretion were determined using Mouse Albumin ELISA Kit (Bethyl Laboratories Inc., Montgomery, TX, USA) and QuantiChrom Creatinine Assay Kit (BioAssay Systems, Hayward, CA, USA) according to the manufacturer's procedures. Mouse urinary ACR was calculated as ACR = urinary albumin/urinary creatinine (*μ*g/mg) as we described before [11].

### 2.6. Kidney Histology and Immunohistochemistry

The kidneys were harvested and fixed in 10% formalin. 5 *μ*m thick sections were stained with periodic acid-Schiff (PAS) reagent. Immunohistochemistry was performed in paraffin sections using a high-temperature-heating antigen retrieval method. Primary antibody used in the present study was proliferating cell nuclear antigen (PCNA, Maixin, Fuzhou, China). After being incubated with the secondary antibody (Proteintech Group, Chicago, IL, USA), 2 *μ*m thick sections were developed with SP immunohistochemical kit (Maixin, Fuzhou, China) to produce a brown product and counterstained with hematoxylin. Histologic evaluation was performed using a Nikon Eclipse E600 microscopy system without knowledge of the identity of the various groups.

### 2.7. Real-Time PCR

Total RNA of kidney samples was extracted using TRIzol (Invitrogen, Carlsbad, CA, USA), according to the manufacturer's instructions. RNA concentrations and purities were quantified using the NanoDrop 2000 Spectrophotometer (Thermo Scientific, Wilmington, DE, USA). cDNA was reverse-transcribed from total RNA with reverse transcription-PCR kit (Takara, Shiga, Japan), according to manufacturer's protocol. Real-time PCR was carried out in the ABI 7300 Real-time PCR system to determine the change in expression of various mRNA levels as described before [12]. The housekeeping gene GAPDH was used as an internal control.

### 2.8. Western Blotting Assay

The kidney tissues were homogenized and the cells were sonicated in RIPA buffer (Solarbio, Shanghai, China). The proteins were electrophoresed on 10% SDS-PAGE gel and transferred onto a polyvinylidene difluoride membrane (Millipore, Billerica, MA, USA). The membranes were blocked with 5% nonfat milk for 1 h and then were incubated overnight at 4°C with the following primary antibodies: PAI-1, intercellular adhesion molecule-1 (ICAM-1, Abcam Inc., Cambridge, MA, USA), Akt, p-Akt, NF-*κ*B, and *β*-Actin. After four times washing with TBST, membranes were incubated with the appropriate secondary antibodies for 1 h at room temperature. Immunoreactive bands were developed by enhanced chemiluminescence after triple washing with PBS-Tween and scanned by an automatic digital gel image analysis system (Tanon-4200, Tanon, Shanghai, China).

### 2.9. Statistical Analysis

*In vivo* and *in vitro* data were collected from at least six animals or at least three separate cell cultures for each group and presented as means ± SD. Comparisons between groups were performed by one-way ANOVA, followed by Tukey's post hoc test. Statistical analysis was performed with Prism 6.0 data analysis and graphing software. Statistical significance was considered as *P* < 0.05.

## 3. Results

### 3.1. RSV Attenuated HG-Induced PAI-1 Expression and Akt Activation *In Vitro*

**Figure 1:**
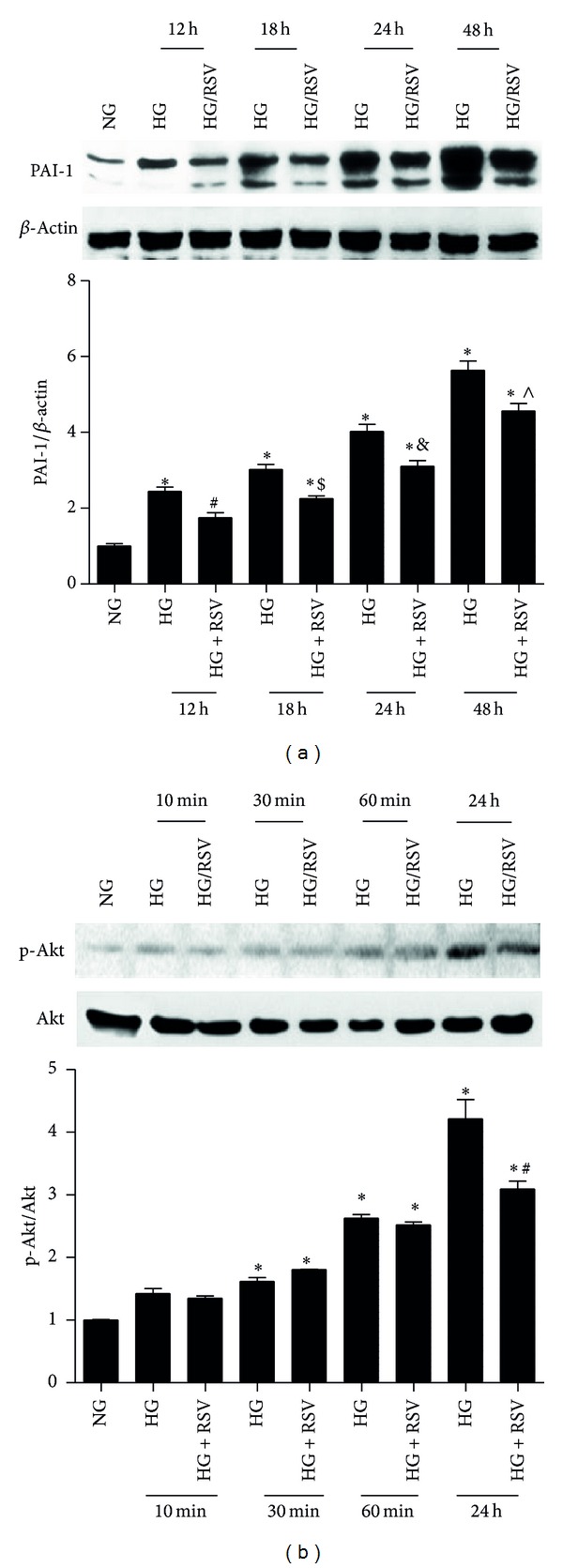
Resveratrol (RSV) attenuated HG-induced PAI-1 expression and Akt activation in rat mesangial cell (RMC). RMCs were cultured in DMEM containing 5.6 mM glucose (NG) or 25 mM glucose (HG) in the absence or presence of RSV (25 *μ*M) for different periods of time, the protein levels of plasminogen activator inhibitor (PAI-1) (a); Akt and p-Akt (b) were detected using Western blotting assay. Results represent the mean ± SD. *P < 0.05 compared with NG,^#^ *P*, ^$^ *P*, ^&^ *P*, and ^∧^ *P* < 0.05 compared with HG in the same time group.

Quiescent RMCs exposed to HG (25 mM) for indicated time were treated with or without RSV (25 *μ*M). After that, cells were harvested for analysis. As shown in Figure 1(a), PAI-1, an inflammation marker, was significantly increased by HG but decreased after RSV treatment from 12 h to 48 h, accompanied by p-Akt/Akt ratio elevation from 10 min to 24 h (Figure 1(b)).

### 3.2. RSV Attenuated HG-Induced PAI-1 Expression and Cell Proliferation *In Vitro*, Which Might Be Akt/NF-*κ*B Pathway Dependent

**Figure 2:**
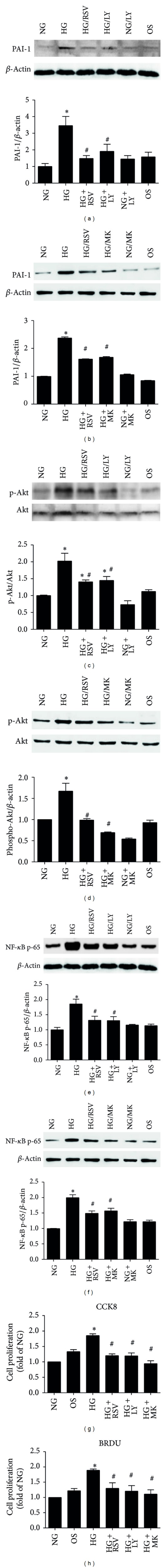
Resveratrol (RSV) attenuated high glucose- (HG-) induced plasminogen activator inhibitor (PAI-1) expression and cell proliferation in rat mesangial cell (RMC), which might be Akt/nuclear factor-kappa B (NF-*κ*B) pathway dependent. RMCs were cultured in DMEM containing 5.6 mM glucose (NG) and 10% FBS. When the confluence reached at 60%–70%, the medium was replaced with NG and 0.2% BSA. 24 h later, the cells were pretreated with 10 *μ*M LY294002, 1 *μ*M MK-2206, or an equal volume of DMSO for 30 min and then incubated for another 24 h with or without HG (25 mM) in the presence or absence of RSV (25 *μ*M). After that, cells were collected and protein levels of PAI-1 ((a), (b)), Akt, p-Akt ((c), (d)), and NF-*κ*B ((e), (f)) were detected using Western blotting assay. Additionally, cell proliferation was examined with CCK8 assay and BrdU incorporation, respectively. Results represent as mean ± SD. *P < 0.05 compared with NG, ^#^ *P* < 0.05 compared with HG.

To determine the relationship between increased PAI-1 expression and Akt activation, the Akt activity inhibitors, LY, and MK were used. After 24 h of treatment, cells were harvested for analysis. As shown in Figure 2, PAI-1 expression (Figures 2(a) and 2(b)) and cell proliferation (Figures 2(g) and 2(h)) were significantly increased in HG group, and these changes were abolished by either RSV or Akt activity inhibitors treatment, suggesting HG-induced PAI-1 over-expression and mesangial cell proliferation through PI3K/Akt signaling pathway. This hypothesis was confirmed by further detecting of p-Akt/Akt ratio and NF-*κ*B, a downstream target of Akt. Similar to the change of PAI-1 and cell proliferation, increased p-Akt/Akt ratio (Figures 2(c) and 2(d)) and NF-*κ*B (Figures 2(e) and 2(f)) protein levels in HG group were also reversed by either RSV or Akt activity inhibitors.

### 3.3. RSV Protected Mice from Diabetes-Induced Kidney Dysfunctional and Structural Changes *In Vivo*

**Figure 3:**
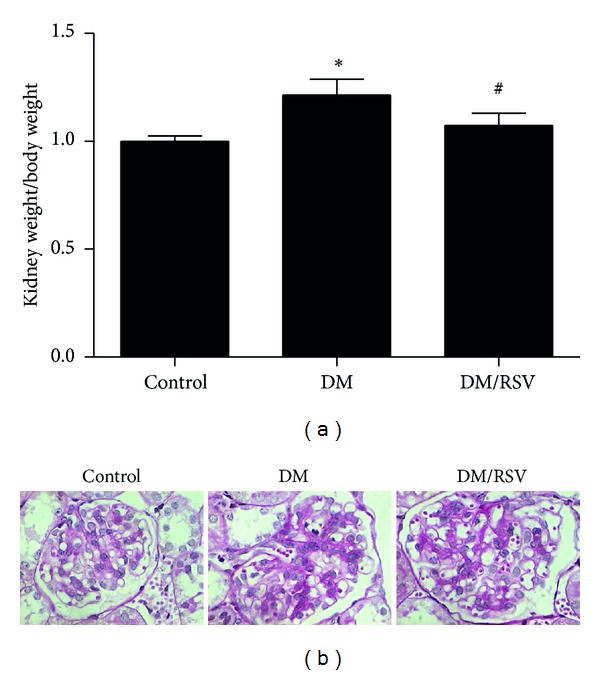
Resveratrol (RSV) protected mice from diabetes-induced renal hypertrophy and structural changes in mice. Kidney weight to body weight ratio (a) of three groups was presented. PAS staining of rat glomeruli sections (×400) was shown as Figure 3(b). Results represent as mean ± SD. *P < 0.05 compared with control group, ^#^ *P* < 0.05 compared with diabetes mellitus (DM) group.

To determine the effects of RSV on the development of diabetes-induced kidney damage, STZ-induced-diabetes mouse model was utilized. After STZ injection, RSV (10 mg/Kg) was given by gavage administration once daily for 12 weeks. At the end of the experiment, mice were killed and blood, urine, and kidney tissue were harvested. RSV-treated diabetes mice developed similar levels of blood glucose, urea nitrogen, and serum creatinine as diabetes mice. However, ACR was significantly decreased in RSV-treated DM group (Table 1). Additionally, we found that mice in DM group developed renal hypertrophy with increased kidney weight to body weight ratio (Figure 3(a)) and increase glomerular area and extracellular matrix (ECM) accumulation (Figure 3(b)), while RSV treatment significantly prevented glomerular enlargement.

### 3.4. RSV Downregulated Akt/NF-*κ*B Pathway in Diabetes Mouse Kidney *In Vivo*

**Figure 4:**
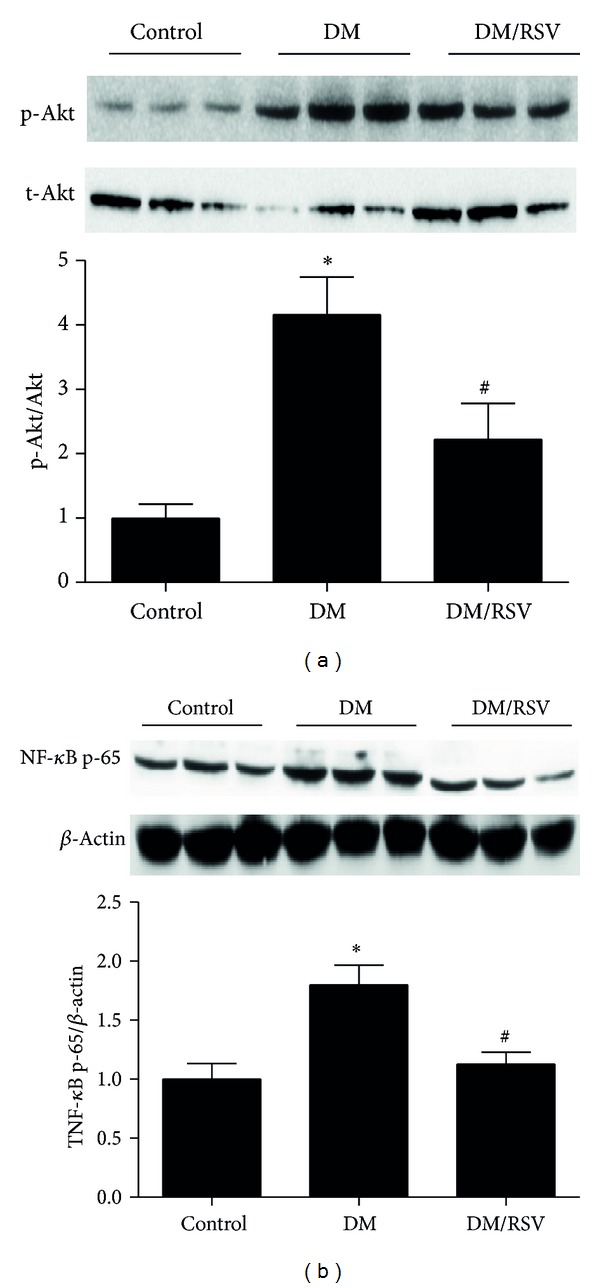
Resveratrol (RSV) downregulated Akt/nuclear factor-kappa B (NF-*κ*B) pathway in diabetes mouse kidney. Western blot analysis was carried out to measure the protein levels of Akt, p-Akt (a) and NF-*κ*B p65 (b). Results represent as mean ± SD. *P < 0.05 compared with control group, ^#^ *P* < 0.05 compared with diabetes mellitus (DM) group.

As shown in Figure 4, kidney p-Akt/Akt ratio (Figure 4(a)) and NF-*κ*B (Figure 4(b)) were significantly increased in DM group but not in RSV-treated DM group. These data were consistence with the *in vitro* results and further confirmed our hypothesis.

### 3.5. RSV Protected Mice from Diabetes-Induced Kidney Inflammation and Cell Proliferation *In Vivo*

**Figure 5:**
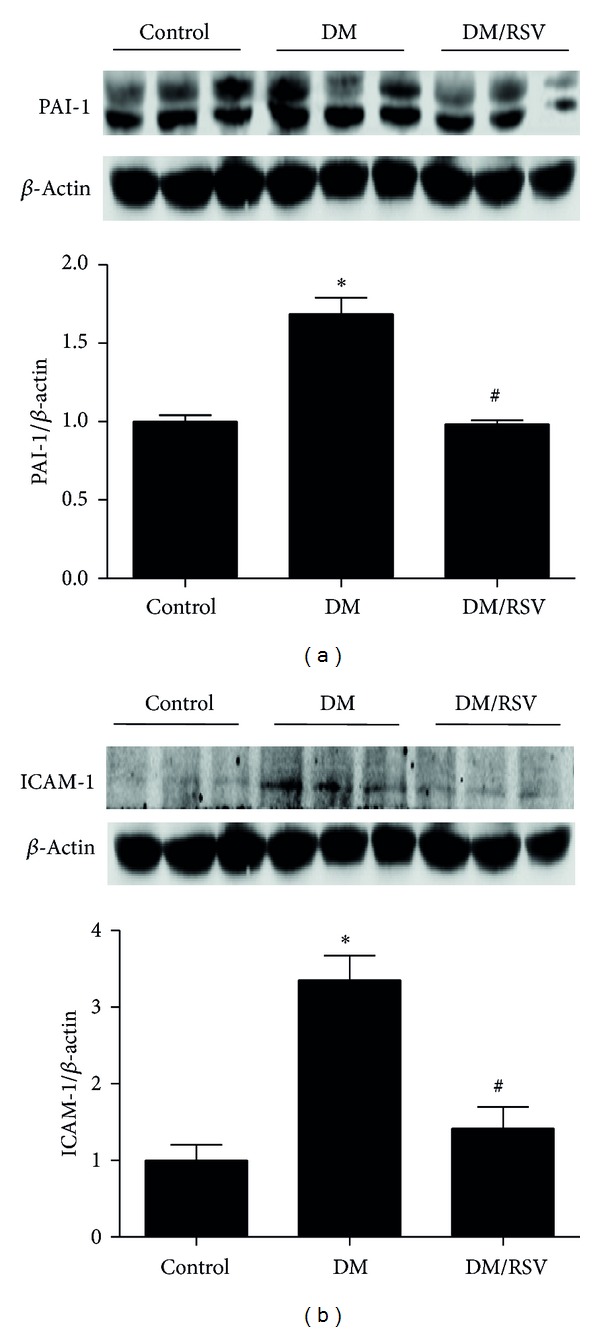
Resveratrol (RSV) protected mice from diabetes-induced kidney inflammation. Western blot analysis was carried out to measure the protein levels of plasminogen activator inhibitor (PAI-1) (a) and intercellular adhesion molecule-1 (ICAM-1) (b). Results represent as mean ± SD. *P < 0.05 compared with control group, ^#^ *P* < 0.05 compared with diabetes mellitus (DM) group.

**Figure 6:**
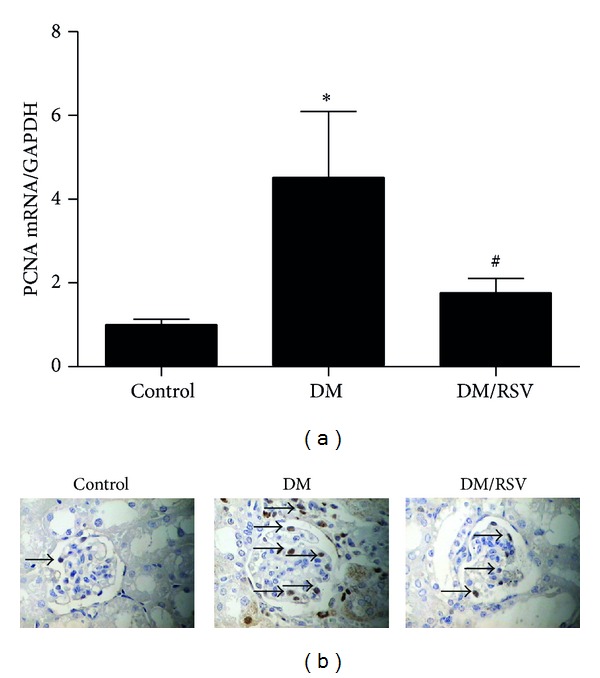
Resveratrol (RSV) protected mice from diabetes-induced mesangial cell proliferation in glomeruli. The proliferating cell nuclear antigen (PCNA) mRNA levels were detected using Real-time PCR (a). The representative images showed PCNA-positive cells (with brown nuclear) in kidney of three groups (×400) (b). Results represent as mean ± SD. *P < 0.05 compared with control group, ^#^ *P* < 0.05 compared with diabetes mellitus (DM) group.

Besides Akt/NF-*κ*B pathway changes, PAI-1 (Figure 5(a)) and ICAM-1 (Figure 5(b)) were also increased in diabetes kidney in the protein levels. In addition, PCNA, a marker of cell proliferation, was also detected. As shown in Figure 6, kidney PCNA mRNA (Figure 6(a)) and the number of positive cells found in glomeruli (Figure 6(b)) were significantly increased in diabetes mice compared with those in control mice. However, these changes were reversed by RSV.

## 4. Discussion

The present study investigated the renoprotective potential of RSV against hyperglycemia-mediated inflammation and mesangial cell proliferation both *in vitro* and *in vivo* and revealed the following innovative findings. Primarily, our data provide confirmatory evidence that RSV treatment may attenuate kidney inflammation and mesangial cell proliferation in diabetes model both *in vivo* and* in vitro*. Secondly, both RSV and Akt activity inhibitors reduced the high glucose-induced upregulation of mesangial cell proliferation as well as PAI-1 and ICAM-1 protein levels, which was Akt/NF-*κ*B pathway dependent.

The alteration of Akt activity in DM takes part in the pathophysiology of diabetic microvascular complications. Studies focusing on Akt in diabetes suggested both decrease and increase in Akt activity in DM [13]. Several studies reported that Akt activity was increased in DN [14, 15], which was consistent with our current result and could be downregulated by RSV. Yet, to the best of our knowledge, such observation has not yet been reported in renal mesangial cells. Furthermore, active Akt is considered as one of physiological activators of NF-*κ*B [13]. Sheu et al. reported that PI3K inhibitors effectively attenuated HG-mediated NF-*κ*B activation in mesangial cells [16]. Consistent with above observations, we found that both RSV and Akt activity inhibitors could inhibit HG-induced NF-*κ*B overexpression in mesangial cells. Taken together, the beneficial effect of RSV on DN might be associated with deactivation of Akt-NF-*κ*B pathway.

Recent studies have established that RSV has protective effects on the development of DN in animals by interacting with different targets, including Akt [8–10, 17–22]. However, the mechanism by which RSV decreases Akt phosphorylation remains not fully understood. In Kim et al.'s study, the PI3K-Akt pathway suppression by RSV was due to the activation of AMPK-Sirt1-PGC-1*α* pathway in kidney *in vivo* [8]. Another study provided a MAPK-Sirt1-PGC-1a independent pathway. Fröjdö et al. claimed that RSV targets class IA PI3Ks by directly binding to the p110*α* and p110*β* catalytic lysine residues of PI3K and consequently inhibits their downstream signaling molecules-Akt [23]. To determine the exact mechanism whereby RSV deactivates Akt activity, further studies are needed.

Increasing data suggest a pivotal role for NF-*κ*B in a variety of pathophysiological conditions in which either inflammation or cell number control is critical events. Most of the current clinical and experimental strategies to reduce the progression of DN, such as renin-angiotensin system inhibitors [24], thiazolidinedione [25], and statins [26], are known to modulate NF-*κ*B [27]. NF-*κ*B promotes the expression of a number of genes involved in inflammation, such as PAI-1 and ICAM-1 [28]. DN is characterized by excessive accumulation of ECM in the kidney. PAI-1 plays an important role in ECM remodeling through increased ECM synthesis as well as decreased ECM degradation [29]. It is also reported that PAI-1 is critically involved in inflammatory responses associated with NF-*κ*B pathway in kidneys from diabetic rats [3]. ICAM-1, which is induced in HG-treated renal mesangial cell through a NF-*κ*B dependent way [4], promotes inflammation by enhancing leukocyte infiltration and is involved in the pathogenesis of DN [30]. Similarly deficiency of ICAM-1 resulted in a substantial decrease in macrophage accumulation in the glomeruli leading in turn to a reduction in glomerular hypertrophy and interstitial fibrosis in ICAM-1 deficient *db/db* mice [31]. In the present study, we provided evidence here that exposure to HG significantly increased the expression of NF-*κ*B and its downstream gene PAI-1. RSV treatment significantly inhibited elevation of these two pathogenic cytokine factors in renal mesangial cells. Furthermore, the administration of Akt activity inhibitors greatly diminished the expression of NF-*κ*B and PAI-1. Similarly, in diabetic mice, RSV treatment improved renal hypertrophy, accumulation of ECM, and albuminuria by downregulating the expression of NF-*κ*B, PAI-1, and ICAM-1.

In recent years, more and more data suggest that NF-*κ*B may play an important role in the control of cell proliferation [32, 33]. It was reported that RSV could inhibit HG-induced renal mesangial cell proliferation through NF-*κ*B pathway [10]. In our current study, we confirmed this point *in vitro*. Meanwhile, we also reported that the Akt activity inhibitors could also inhibit HG-induced renal mesangial cell proliferation and NF-*κ*B activity. As indicated above, the HG-induced Akt activity was downregulated by RSV and Akt activity inhibitors. Based on these, we demonstrated that RSV might be through Akt/NF-*κ*B pathway to inhibit renal mesangial cell proliferation. Consistent with this view, in our *in vivo* study, we observed that the number of PCNA-positive mesangial cells in glomerulus and PCNA mRNA level in DM group were increased compared with those in control group, which was supported by a previous study [14]. These changes could be attenuated by RSV treatment. To our knowledge, this is the first* in vivo* evidence that RSV protects DN by reducing mesangial cell proliferation.

## 5. Conclusion

**Figure 7:**
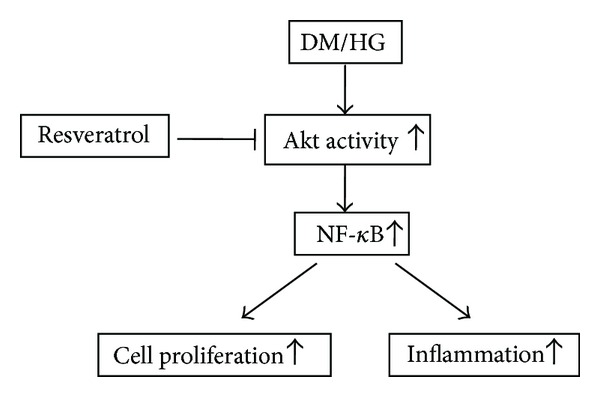
Schematic representation of proposed intracellular signaling leading to renoprotective potential of resveratrol against hyperglycemia-mediated inflammation and mesangial cell proliferation in diabetes.

Collectively, as indicated in Figure 7, our results showed augmented p-Akt/Akt in HG-treated mesangial cells, and such induction seemed to be attenuated by RSV and Akt activity inhibitors. Furthermore, in our *in vivo* study, we demonstrated that p-Akt increased in whole kidney lysates from 3-month STZ-induced diabetes mice and RSV treatment downregulated p-Akt expression. Since active Akt is one of physiological activators of NF-*κ*B [13], it is possible that RSV inhibited NF-*κ*B activity via suppression of Akt activity and, consequently, attenuated inflammation and renal mesangial cell proliferation to protect DN.

## Figures and Tables

**Table 1:** The effects of RSV on biochemical parameters in diabetes mice.

	Control (*n* = 6)	DM (*n* = 8)	DM/RSV (*n* = 8)
Blood glucose (mg/dL)	167.59 ± 19.20	360.63 ± 86.32*	309.49 ± 76.87*
Blood urea nitrogen (mg/dL)	18.42 ± 2.62	43.81 ± 10.16*	37.69 ± 8.69*
Plasma creatinine (mg/dL)	0.22 ± 0.04	0.29 ± 0.03*	0.25 ± 0.04*
ACR (*μ*g/mg)	38.4 ± 5.3	114.6 ± 67.2*	62.2 ± 19.6^#^

Notes: values were expressed as means ± SD (*n* =  6~8 per group). *P < 0.05 versus control group; ^#^ *P* < 0.05 versus DM group. RSV: resveratrol; DM: diabetes mellitus.
